# Quantitative Analysis of Tumor-associated Tissue Eosinophilia in Recurring Bladder Cancer

**DOI:** 10.7759/cureus.3279

**Published:** 2018-09-10

**Authors:** Hristo Popov, Ivan S Donev, Peter Ghenev

**Affiliations:** 1 General and Clinical Pathology, Forensic Medicine and Deontology, Medical University, Varna, BGR; 2 Clinic of Oncology, St. Marina University Hospital Varna, Varna, BGR; 3 General and Clinical Pathology, Forensic Medicine and Deontology, Faculty of Medicine, Varna, BGR

**Keywords:** bladder cancer, recurrence, stroma, eosinophils

## Abstract

The relatively high incidence of recurrence of bladder cancer is a serious problem in clinical practice. At present, there are no objective microscopic criteria for evaluation of the tendency for local relapse. Besides the phenotypic properties of the tumor parenchymal cells, possible signs in regard to recurrence could also be derived from the peculiarities of the tumor stroma. The stromal reaction, manifested by inflammatory infiltration in the tumor is considered to influence the biological behavior of tumors. Also, a relationship has been reported between the number of eosinophils and the survival of patients. The aim of the present study is to analyze tumor-associated tissue eosinophilia (TATE) and to compare TATE density in the initial foci of age and gender-matched 156 cases of recurrent and non-recurrent bladder cancers; the tumors that have relapsed within six months after removal and contained statistically significant greater numbers of eosinophils in primary cancer sites. These results suggest that TATE may be one of the probable prognostic signs for local relapse of urothelial cancer.

## Introduction

Bladder cancer is a common urologic malignancy and has one of the highest recurrence rates [1]. Increasingly high morbidity rates impose a search for more relevant prognostic and predictive markers for tumors in stage pTa and pT1. Histologically, there are no convincingly objective criteria for assessing the tendency of local recurrence. In this regard, possible signs may be derived from some stromal characteristics, such as the presence of eosinophilic leukocytes, mast cells, lymphocytes, plasma cells, fibroblasts and myoepithelial cells. The classical understanding of the role of eosinophilic leucocytes is associated with allergic reactions and certain types of infections [2]. The presence of eosinophils in cancer has been established in some locations and malignant classes, despite some contradictory reports [3-5]. Tumor-associated tissue eosinophilia (TATE) may be associated either with good or poor patient prognosis [6-7].

Some studies support the opinion that eosinophils may have a role in tumor initiation [7]. In colorectal cancer, hematologic tumors, gastric cancer, lung cancer, gynecologic malignancies, and breast cancer, quantitative assessment of eosinophils in tumor tissue is proposed as one of the possible aspects of the histological evaluation, correlating to the patient prognosis [3,5,8-18].

The aim of the present study is to evaluate TATE in primary foci of recurrent and non-recurrent urothelial carcinoma of the urinary bladder.

## Materials and methods

A total of 156 cases of urothelial carcinoma were studied on the primary site formalin-fixed parathion embedded tissue sections, stained with hematoxylin and eosin (H&E). Out of them, 78 had a disease recurrence within a period of six months. Their morphological features were compared to a control group of age and sex-matched 78 cases without recurrence for the same period of time. We examined the density of eosinophilic leukocytes per square millimeter of tissue surface.

We performed morphometric analysis by using digitally scanned slides via Leica Aperio ScanScope AT2 device (Aperio Technologies, Vista, CA) visualized on ImageScope V12.1.0.5029 (Aperio Technologies, Vista, CA).

Statistical analysis was carried out with SPSS Statistics v.23 using descriptive statistics. Categorical features were summarized with frequencies and percentages. The Mann–Whitney U test and Pearson correlation were used for comparison and estimation of correlations between the density of eosinophilic leukocytes in the stroma of non-recurrent and recurrent bladder cancer. Specificity and sensitivity of density of eosinophilic leukocytes in stroma for distinguishing recurrent disease patients (RP) from non-recurrent disease patients (NP) at three years of follow-up were evaluated with receiver operating curve (ROC) analysis. Diagnostic accuracy of biomarkers was also determined by obtaining the largest possible area under the curve (AUC) in ROC analysis. Kaplan-Meier survival curves and the log-rank test were used to compare the survival differences between groups. Although our study was not powered enough to compare different subgroups, hazard ratios (HRs) and corresponding 95% confidence intervals (CIs) were calculated by Cox regression models. Two-tailed p-values (<0.001) were considered as significant.

## Results

**Figure 1:**
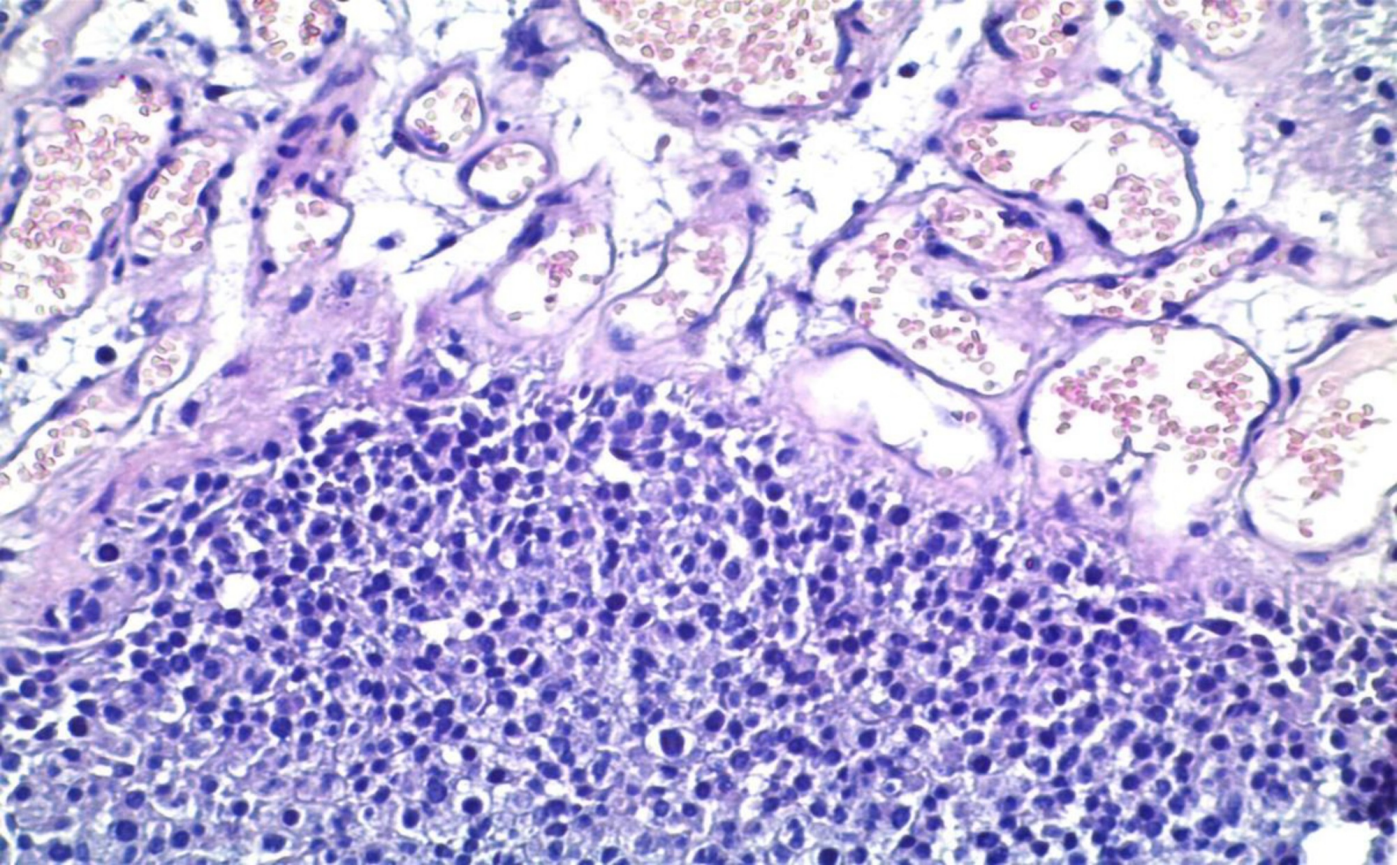
Case number 2: a 70-year-old male with high-grade urothelial carcinoma The average number of eosinophils per square millimeter, in this case, is zero. Hematoxylin and eosin, original magnification x200.

**Figure 2:**
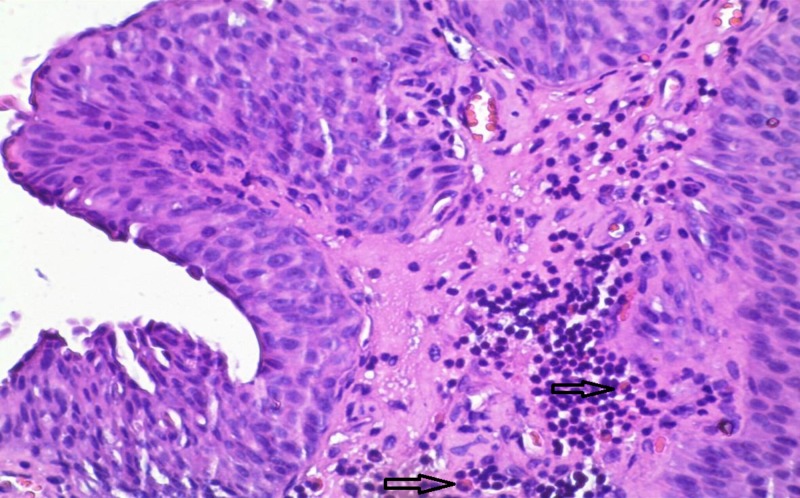
Case number 21: a 38-year-old male with high-grade urothelial carcinoma The average number of eosinophils (arrows) per square millimeter, in this case, is 8.93. Hematoxylin and eosin, original magnification x200.

**Figure 3:**
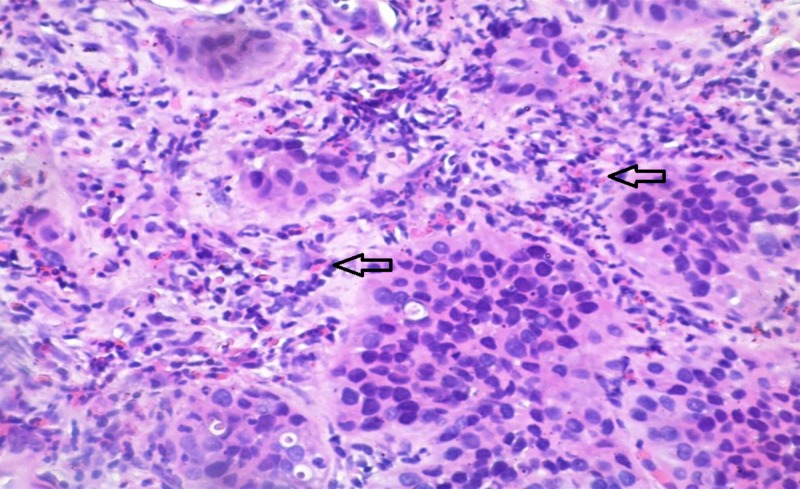
Case number 15: a 67-year-old male with high-grade urothelial carcinoma The average number of eosinophils (arrows) per square millimeter, in this case, is 35.21. Hematoxylin and eosin, original magnification x200.

In the present study, eosinophilic leukocytes were present in the stroma of 66 tumors (42.3%), either as scattered single cells, small groups of cells, or larger clusters. The Mann–Whitney test showed that there are significant differences in the number of eosinophils in the RP and NP group. TATE varied from entirely lacking eosinophilic leukocytes to moderately present and to very high density (Figures 1-3).

**Figure 4:**
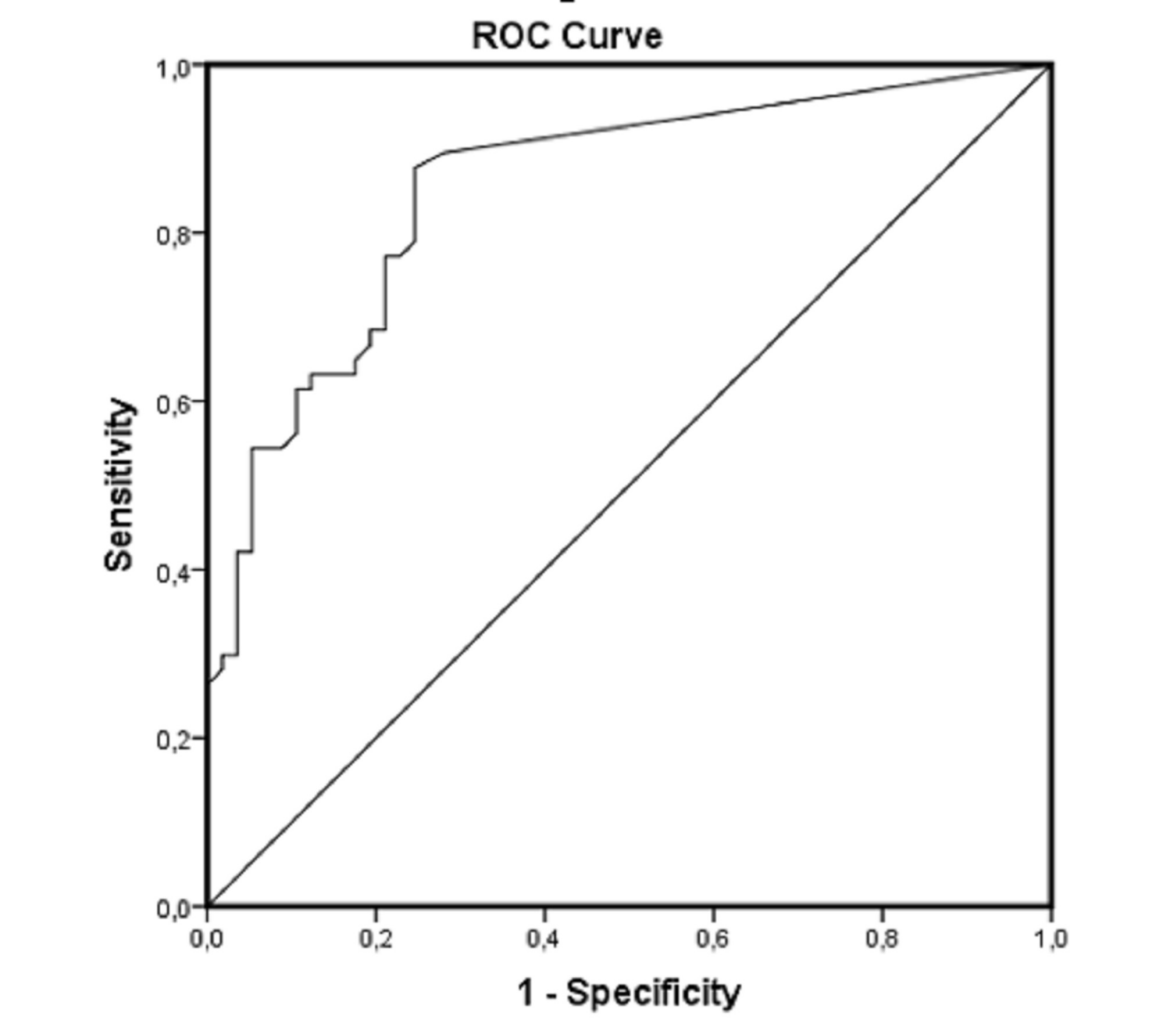
Receiver operating curve (ROC); AUC = 0.853, 95% CI: 0.783–0.924, p < 0.001 AUC: area under curve; CI: coefficient interval

The density of eosinophilic leukocytes in the tumor stroma varied severely between the RP and NP groups (AUC = 0.853, 95% CI: 0.783–0.924, p < 0.001). At the optimal cutoff values of number of eosinophils, the sensitivity was 75.4% and specificity was 78.9% (Figure 4).

**Figure 5:**
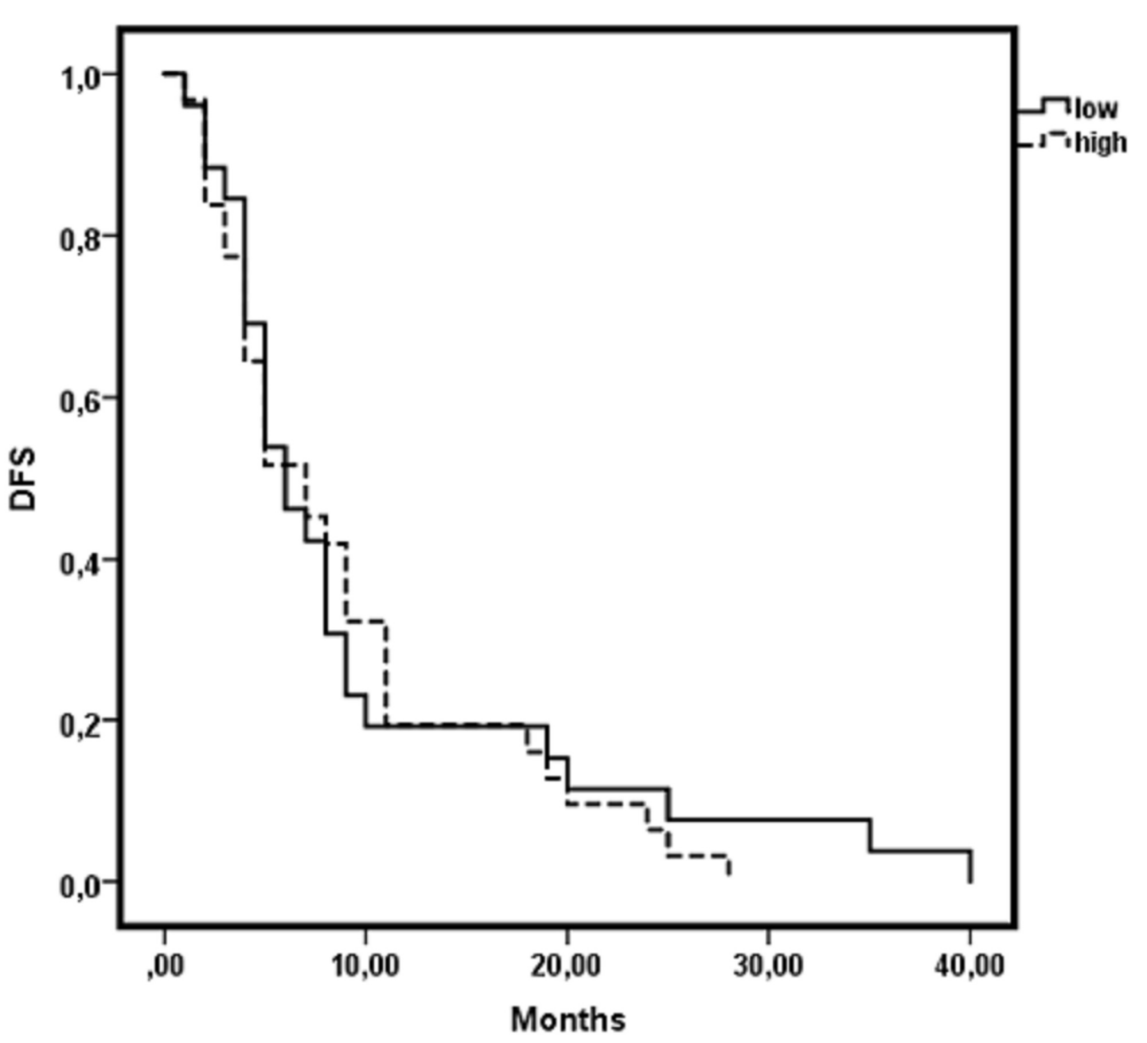
Kaplan-Meier curve: no correlation between the number of eosinophils and disease-free survival (DFS)

Disease-free survival (DFS) was calculated as the time from the date of surgery to the date of documented recurrence of the disease. Cancer patients with a high number of eosinophils had no significant differences in DFS when compared with those with a low number of eosinophils (Figure 5).

The quantitative assessment of TATE in the tumor stroma shows statistically significant higher TATE in primary urothelial cancer, with a tendency to recur.

## Discussion

The present study was designed to evaluate the significance of TATE in the stroma of urothelial cancer. We focused on one of the most peculiar features – the relatively high incidence of local progression as recurrence after surgical removal. The quantitative assessment of TATE in the tumor stroma shows the statistically significant higher density of eosinophils in primary urothelial cancers tending to recur and no influence on DFS. In a similar study of colorectal cancer (in squamous cell cancer with different location), high grades of TATE were associated with the lesser tendency of tumor metastasis.

Increased number of eosinophils may result from either an enhanced recruitment mediated by chemotactic mediators or a decreased apoptosis of eosinophils in the local tissue or both. The exact role of eosinophils in carcinogenesis - tumor transformation and/or progression is not quite clear.

Mature human eosinophils contain numerous highly basic and cytotoxic granule proteins, such as major basic protein, eosinophil peroxidase, eosinophil cationic protein, and eosinophil-derived neurotoxin. Their activity is related to the release of these substances and basement membrane damage by eosinophil-derived cation proteins and peroxidase [9,19-24]. These products interfere in a variety of cellular processes involved in tissue remodeling and tumor behavior [9]. The presence of eosinophils in different amounts within the stroma of 66 bladder cancers (42.3%) is obviously due to the action of potent eosinophil chemoattractants, such as eotaxin, platelet activating factor, C5a, interleukin-5, and immunoglobulin E (IgE) [12-13,18]. Some of these have been found in cancer patients - either in the tumor tissue [1] or in the blood [4]. Results from studies in other cancer locations suspect procancer effects of eosinophils via activation on stromal fibroblasts and angiogenesis [6,20]. In addition, eosinophils are capable to contact directly with tumor cells and trigger them into the apoptotic pathway. Collectively, TATE has been reported to be associated with a favorable prognosis for patients with esophageal squamous cell carcinoma and prostate carcinoma. Evidence derived from experimental models [19] also suggests the antitumor potential of TATE. However, up to date, the prognostic role of TATE in cancer patients remains conflicting rather than conclusive, given the inconsistent results from cancer of different origins

To the best of our knowledge, neither of these cellular interactions have been thoroughly studied in bladder cancer. Although most of the studies in other cancer location suggest a beneficial effect of TATE, the present results reveal reliable data for association of eosinophils in local recurrence of urothelial bladder cancer.

Keeping in mind the different significance of TATE, in regard to their location – both intra- versus peritumoral and in different primary tumors, the most probable explanation seems to be that besides their direct interaction with tumor parenchymal and stromal cells, eosinophils have the potential to modulate the functions of other immune cells. Nevertheless, there is still a long way to understand the exact mechanism of the effect of eosinophils in cancer.

At least in urothelial bladder cancer, it is tempting to suggest that TATE might be regarded as a possible prognostic factor for local recurrence. So far, the evaluation of eosinophils represents a promising and readily assessable tool, and should therefore routinely be commented on in the pathology report.

## Conclusions

The quantitative assessment of TATE in tumor stroma shows statistically significant higher TATE in primary urothelial carcinomas tending to recur. Although these findings need further explanation, it is tempting to suggest that TATE might be regarded as a possible prognostic factor for the local recurrence of bladder cancer.
